# Royal Jelly Promotes Ovarian Follicles Growth and Increases Steroid Hormones in Immature Rats

**DOI:** 10.22074/ijfs.2018.5156

**Published:** 2017-10-14

**Authors:** Elham Ghanbari, Mohammad Rasool Khazaei, Mozafar Khazaei, Vahid Nejati

**Affiliations:** 1 Fertility and Infertility Research Center, Kermanshah University of Medical Sciences, Kermanshah, Iran; 2 Department of Histology and Embryology, Faculty of Science, Urmia University, Urmia, Iran

**Keywords:** Fertility, Immature Rats, Ovary, Royal Jelly

## Abstract

**Background:**

Royal jelly (RJ) is a complementary diet widely prescribed by traditional medicine specialists for treatment of in- fertility. The aim of present study was to evaluate the effects of RJ on a set of reproductive parameters in immature female rats.

**Materials and Methods:**

In this experimental study, thirty two immature female rats (30-35 g) were divided into four groups (n=8/group): three experimental groups and one control. The experimental groups received 100, 200 and 400 mg/kg/body weight doses of RJ daily for 14 days, and the control group received 0.5 ml distilled water interaperito- nealy (i.p). The treated rats were sacrificed and their ovaries were dissected for histological examination. The serum levels of ovarian hormones, nitric oxide (NO) and ferric reducing antioxidant power (FRAP) were evaluated, and the ratios of the ovarian and uterine weight to body weight were calculated. One-way ANOVA was used for data analysis.

**Results:**

The body weights were significantly different (P=0.002) among the rat groups, with an increase in all RJ treated animals. Uterine and ovarian weights and the serum levels of progesterone (P=0.013) and estradiol (P=0.004) were significantly increased in experimental groups compared to the control group. In addition, a significant increase in the number of mature follicles and corpora lutea (P=0.007) was seen in RJ recipients compared to the controls. A significant increase in the serum levels of FRAP (P=0.009) and a significant decrease in NO level (P=0.013) were also observed.

**Conclusion:**

RJ promotes folliculogensis and increases ovarian hormones. This product can be considered as a natural growth stimulator for immature female animals.

## Introduction

Puberty is the underlying reproductive process, in which several maturation changes occur in the body to promote adult phenotype. The onset of puberty is sensitive to the amount of energy reserves of the organism and nutritional status and it is associated with the dynamic dialogue between the environment and genes (1). In females, estrogens have a key role in the differentiation, growth and function of the reproductive organs. They are identified as the principal hormones in regulation of the female reproductive system (2). Royal jelly (RJ) is the most important apiary product, secreted by 5 to 15 day old worker honeybees (3). It possesses several health promoting properties such as antitumor effect, antioxidant activity, and improvement of menopausal symptoms and infertility (4). There are four unsaturated fatty acid compounds in RJ (10H2DA, 10HDA, 2DEA and 24MET) that showed estrogen receptors’ (ERs’) β-binding activity. These compounds exhibited estrogenic effects mediated through interactions with ERs, leading to alterations in gene expression and cell proliferation (5). Nonetheless, RJ has been shown to inhibit the harmful effects of exogenous estrogen on male reproductive tract (6). 

Previous study showed that RJ treatment increased plasma progesterone levels in sheep (7). RJ could also be effective in ameliorating pregnancy rate and the lambing rate in Awassi ewes (8). These data prompted us to study the effect of RJ on puberty and fertility parameters in immature female rats. To our knowledge, the mechanism of action of RJ on reproductive function is still unknown. It may exert its effects via changing hormonal secretions or by containing hormone-like compounds. Other studies have shown that treatment of ovine oocytes with 10 mg/mL of RJ during *in vitro* maturation (IVM) increases oocyte and nuclear maturation rate, fertilization rate and blastocyst formation, which might be due to increased activity of antioxidant enzymes in both oocyte and cumulus cells (9). It has been reported that therapy with bee honey plus RJ may be an effective approach to treat infertility due to asthenozoospermia (10). 

It has been suggested that over-nutrition before puberty, as well as excessive weight gain might affect the maturation of reproductive system and induce an early onset of puberty (11). It should be noted that during the final oocyte maturation stage, alterations at levels of proteolytic enzymes, cytokines, prostaglandins and nitric oxide (NO) lead to an increase in the level of reactive oxygen species (ROS), thereby inducing ovarian blood flow and helping follicular rupture (12). It has also been suggested that excessive amount of follicular ROS may disrupt the antioxidant defense system, leading to oocyte injury (13). Interestingly, abnormal ROS levels might facilitate various pathological processes, specifically in the ovary and uterus, causing decreased pregnancy maintenance hormones and luteal regression (14,15). The antioxidant effect of RJ has been widely examined and confirmed on the male reproductive system (15), but there is no data regarding the effect of RJ on immature female reproductive tract. The present study is designed to investigate the effects of RJ on ovarian maturation as well as changes in related parameters in immature female rats. 

## Materials and Methods

In this experimental study, 32 immature female Wistar rats aged about 25 days (30-35 g) were used for our study. They were maintained under standard environmental conditions of light (12 hour cycle), temperature (24 ± 2°C) and humidity (30-70%), and were fed with a standard laboratory diet and water ad libitum. The protocol of this study was approved by Animal Care and Use Committee at Kermanshah University of Medical Sciences. In addition, all animal procedures were in agreement with the guidelines of the Ethical Committee for research on laboratory animals at Kermanshah University of Medical Sciences (KUMS.REC.1395.168). 

RJ was gathered from six colonies of Iranian honeybees (Apis mellifera) at the apiary of Urmia region, North-West of Iran. RJ samples were harvested in sterile bottles at 68-72 hours after bee larvae were transferred into the queen cell. Different doses of RJ were dissolved in 0.5 ml distilled water; this mixture was prepared freshly every day. 

### Experimental design

The rats were randomly classified into 4 groups (n=8): three groups received 100, 200 and 400 mg/kg/day doses of RJ interaperitonealy (i.p). These doses were chosen according to previous studies (16,17).The fourth group was our control, which received distilled water for 14 days (i.p) (18). On day 14, the rats were weighted and euthanatized by chloroform inhalation in a closed chamber. Blood was collected by cardiac puncture and centrifuged at 2500 rpm for 15 minutes at 4ºC, and the collected serum was stored at -20ºC for the hormones assay (estradiol and progesterone), FRAP, and NO assay. 

### Hormonal assay

The levels of estradiol and progesterone in the collected sera were determined by ELISA kit (DRG International, GmbH, USA), and the reagents to perform the assays were purchased from GBC (General Biological Corporation, Hsin Chu, 30 077, Taiwan, R.O.C). The concentrations of these hormones were assayed via absorbance reading to Microtiter (well reader LabSystems Multiskan RC, 351, FIN-0 0881, Helsinki, Finland) at 450 nm. 

### Ferric Reducing Antioxidant Power assay

The total antioxidant capacity in the collected sera was evaluated by Ferric Reducing Antioxidant Power assay (FRAP) method. Briefly, 150 μl serum was mixed with 1.5 ml of fresh FRAP reagent (10 mM 2, 4, 6- Tripyridyl-s-Triazine, 20 mM Fecl3, 6H2O solution and 300 mM acetate buffer pH= 3.6), and incubated at 37˚C for 10 minutes. Absorbance at 593 nm was then measured using a spectrophotometer (Pharmacia, Novaspec II, Biochrom, England) and was compared with a standard curve constructed with known concentrations of FeSO_4_ 7H_2_O. Results were expressed in μM (19).

### Nitric oxide assay

The serum levels of NO were determined colorimetrically by Griess method, which includes the conversion of nitrate to nitrite. Griess reagent facilitates the conversion of nitrite to a deep pink azo substance (20). Briefly, equal volumes of Griess reagent and serum samples were mixed and incubated at room temperature for 30-45 minutes. Next, the absorbance rate was determined at 540 and 630 nm using ELISA reader (STAT Fax 100, USA). 

### Ovarian histology evaluation

The ovaries and uteri were dissected and cleaned from fatty tissues and were weighed afterwards. The ovaries were fixed in 10% formalin, sectioned at 6 μm thickness and stained with hematoxylin and eosin (H and E) for histological analysis. The five largest sections of each ovary were used for follicular and corpora lutea investigation. Histopathological examination was performed to measure the effects of RJ on the mean number of primary, secondary, graffian follicles and corpora lute. Samples were evaluated for histological changes under an optical microscope and images were captured for further analysis using an image processing software (Motic 2000) (21). 

### Statistical analysis

All data were analyzed by SPSS software (version 18) and presented as mean ± SE. There were no outliers, and the data were normally distributed for each group, as evaluated by box plot and Shapiro-wilk test, respectively. The assumption of homogeneity of variance was also evaluated (P value for Levene’s test was not significant). Comparison of data between the groups was performed by one-way ANOVA followed by post-hoc Tukey test with significance level of P<0.05. 

## Results

### Whole body, ovarian and uterine weight changes

**Fig.1:**
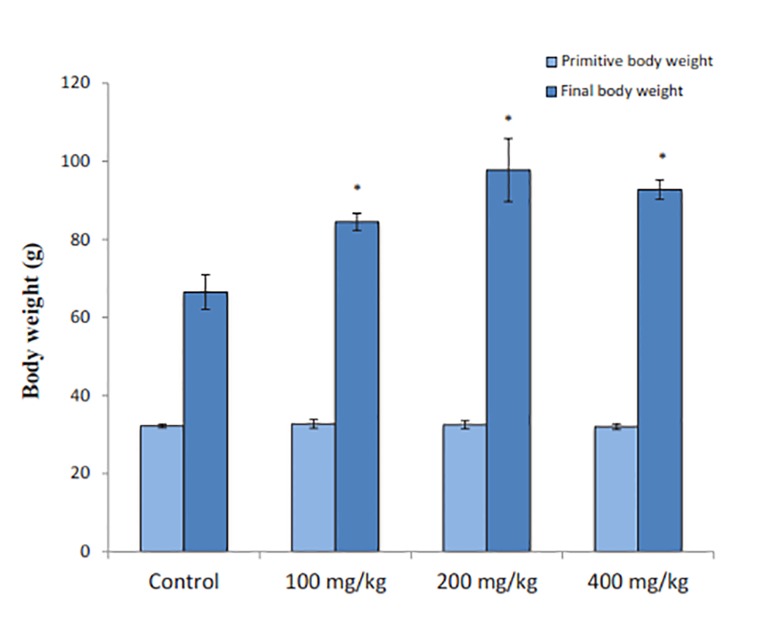
Effect of royal jelly (RJ) on body weight (first and final weight). Values represent mean ± SE for 8 rats in each group. *; P< 0.05 shows significant difference from control group.

**Fig.2:**
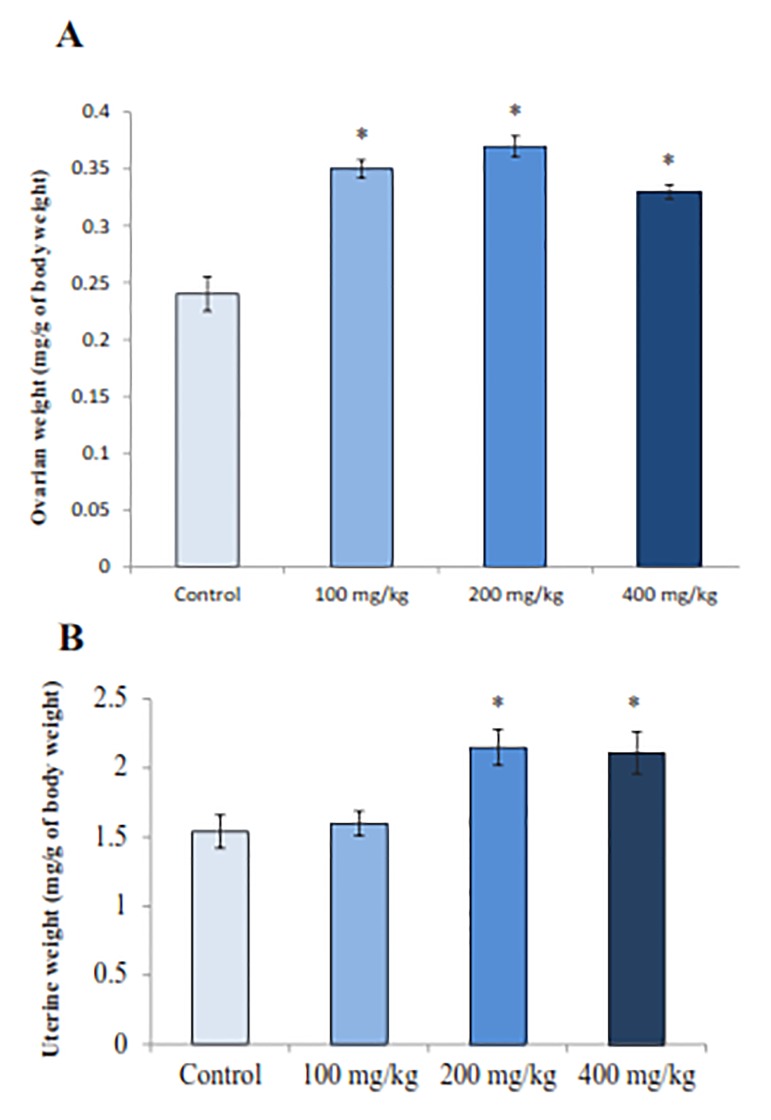
Royal jelly (RJ) effect on the ovarian and uterine weights. A. Histogram of ovarian weights and B. Histogram of uterine weights. *; Values significantly different at P<0.05 from control group (ANOVA). Each histogram represents the mean ± SE of the values for 8 rats.

Administration of different RJ doses to immature female rats for 14 days resulted in a significant increase (P=0.002) in body weights compared to the control rats. On the 1^st^ day, there was no significant difference (P=0.9) between all groups in the mean body weight (Fig .1). All three doeses of RJ significantly increased the ovarian (P=0.002) and uterine (P=0.007) weights in comparison to controls (Fig .2A,B). 

### Ovarian hormones

**Fig.3:**
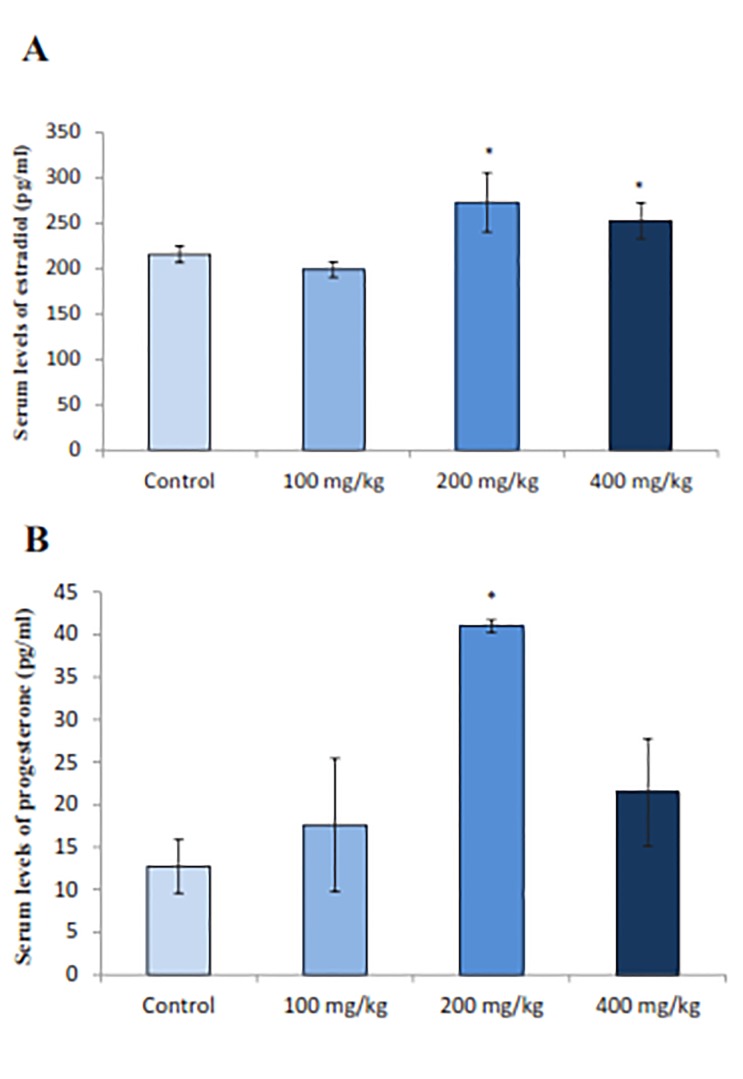
Effect of royal jelly (RJ) on the serum levels of estradiol and progesterone. Values significantly different respectively at P<0.05 from control group (ANOVA). A. Histogram of serum levels of estradiol and B. Histogram of serum levels of progesterone. Each histogram represents the mean ± SE of the values for 8 animals. *; P<0.05 represents significant difference from control group.

The effect of RJ on production of ovarian hormones was a significant rise in the serum estradiol levels in the groups treated with 200 and 400 mg/kg doses of RJ (P=0.004) compared to the control group (Fig .3A). Serum estradiol values for doses 100, 200 and 400 mg/kg of RJ were 198.7 ± 8.3, 273.0 ± 32.6 and 252.7 ± 39.29 (pg/ml), respectively, whereas it was 150.0 ± 9.28 (pg/ml) for the control group. Similarly, RJ treatment led to a significant increase (P=0.013) in serum progesterone levels. The progesterone concentration was measured to be 12.73 ± 7.8, 17.62 ± 7.82, 41.00 ± 1.44 and 21.45 ± 3.64 (pg/ ml) in control, 100, 200 and 400 mg/kg doses of RJ, respectively (Fig .3B). 

### Nitric oxide and Ferric Reducing Antioxidant Power levels

**Fig.4:**
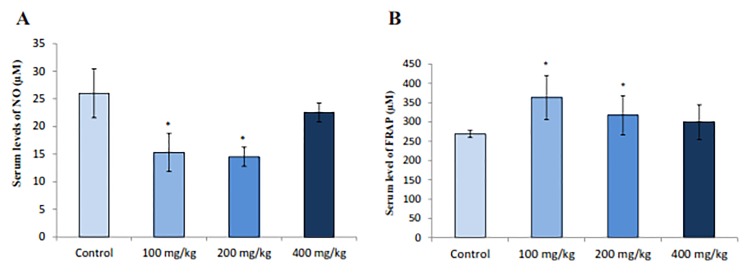
Effect of royal jelly (RJ) on serum NO and FRAP in immature female rats. A. Histogram of serum levels of NO and B. Histogram of serum levels of FRAP. Data represent the mean ± SE (n=8 for each group). NO; Nitric oxide, FRAP; Ferric reducing antioxidant power, and *; P<0.05 indicates significant difference from control group.

NO concentration was measured in the rat sera and the results showed that RJ decreased NO levels in immature rats significantly (P=0.013) (Fig .4A). Also, significant increase (P=0.009) in serum levels of FRAP was observed in normal rats treated with doses 100 and 200 of RJ compare to control group (Fig .4B) 

### Ovarian structure 

**Fig.5:**
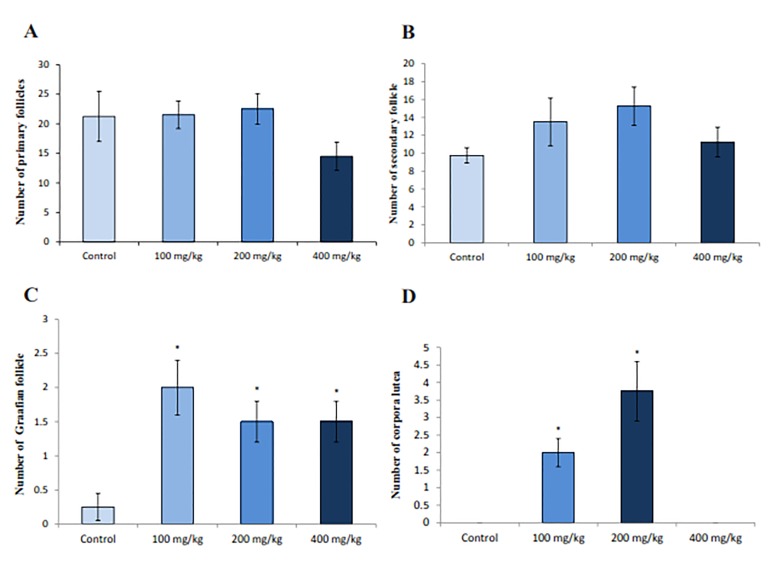
Effect of royal jelly (RJ) on the number of follicles and corpora lutea in ovarian cortex. A. Histogram of the number of primary follicle, B. Histogram of the number of secondary follicles, C. Histogram of the number of graffian follicles, and D. Histogram of the number of corpora lutea. *; P<0.05 shows statistically signiﬁcant difference from control group.

**Fig.6:**
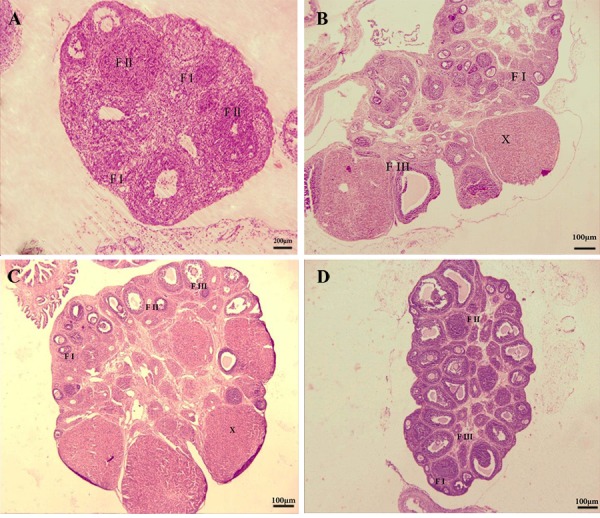
Representation of histological study on immature female rat ovaries. A. Ovaries from the control (magniﬁcation: ×10, scale bar=200 μm), B. Treated with 100 mg/kg, C. 200 mg/kg royal jelly (RJ), and D. 400 mg/kg of RJ. The ﬁgures show primary, secondary and graffian follicles and also observed the corpora lutea (magniﬁcation: ×4, scale bar=100μm). Primary follicles (F I), the secondary follicles (F II), the antral follicles (F III) and Corpora lutea (X).

Microscopic observations of the treated rat ovaries showed an increase in the mean numbers of secondary, antral and graffian follicles when 100 and 200 mg/kg doses of RJ were administered. There was also a significant increase (P=0.007) in the number of corpora lutea of the rats treated with 100 and 200 mg/kg doses of RJ compared to the control rats (Fig .5,6). 

## Discussion

In this study, administration of different doses of RJ to immature female rats led to remarkable increase in the serum steroid hormones, and in body, uterine and ovarian weights. These results suggest that, as a consequence of its estrogenic effects, RJ may have beneficial reproductive influence on both ovarian and uterine structures (22). Pervious studied have shown that consumption of dietary estrogenic compounds in immature rodents promotes female sexual maturation (23). 

Ovarian steroids have pivotal roles in fertility, maturation and growth of reproductive organ. It seems that RJ components exert their effects by binding to estrogen receptors. Previous reports have shown that four main isolated components from RJ bind preferentially to estrogen receptor β rather than estrogen receptor α in immature rats (5). In addition, different bioactive compounds in RJ have been identified that promote cell growth, cell survival and cell differentiation in insects (24). Also, a RJ protein (350 kDa), which seems to be a stimulator of reproductive system development has been reported (25), which might be the growth promoting factor leading to the weight changes that we observed in the rat ovaries as a result of RJ treatment. 

The weight gains that were noticed following RJ administration in our study may be related to high amino acid and protein contents of RJ, which might be involved in metabolic pathways for tissue synthesis and body growth. On the other hand, royalactin, a 57-kDa protein in RJ, induces the development of honey bee larvae to queens via epidermal growth factor receptor-mediated signaling pathway, as well as elevating body size and ovarian development (26). Additionally, it has been shown that treatment with RJ may beneficially impact ovulation rate, which might be associated with an increase in progesterone levels in luteal phase (7). 

Kridli et al. (27) have reported a minor decrease in interval to the onset of estrus, as the effect of RJ administration which can be associated with the stimulating properties of RJ on follicular development and growth. Similar to our findings, other studies suggest that RJ increases the development and growth of follicle resulting in estradiol secretion required to stimulate behavioral estrus, luteinizing hormone (LH) surge and ovulation (7). To assess the dose of RJ required for an optimal effect, we selected three different doses of RJ according to our previous experiments (15) and other related studies (16,28,29). Our data showed that 200 mg/kg of RJ is an efficient dose in our study and exerts significant differences in many studied parameters. 

Since we were interested in studying the effects of RJ administration in immature rats, we chose 25 day old rats and treated them for a period of 14 days to complete our study before complete rat maturation. In terms of maturation, our data showed that treatment with 200 mg/kg RJ increased the mean number of secondary, antral and graffian follicles and corpus luteum, indicating that RJ exerts a stimulating effect on folliculogenesis and ovulation. 

Recent studies have suggested that ovarian/oocyte NO production plays a key role in oocyte meiotic maturation and ovulation. Also, at the time of ovulation, NO may function as a signal for somatic cells of the follicle wall, which are necessary for ovulation (30). In the present study, however, the serum NO levels in immature female rats significantly decreased with administration of RJ. This reduction of serum NO might be the result of increasing serum levels of FRAP. These observations are consistent with the findings of other researchers, who have stated that paclitaxel administration increases NO level, while treatment with RJ (50, 100 and 150 mg/kg) is able to significantly protect this reduction of NO level (29). 

In female animals changes in the physiological concentration of ROS possess a pivotal role in the reproductive functions, including oocyte maturation, ovarian steroidogenesis, folliculogenesis, luteolysis and ovulation. In addition, cumulus cells, follicular fluid and oocytes are endogenous sources of free radicals, such as NO, in female reproductive tissues (31). Although free radicals possess many physiological outcomes, higher production of such compounds (e.g. NO, superoxides and H_2_O_2_) may lead to an elevated risk of ovarian pathology that would probably intensify under decreased antioxidant defense system (32). The possible antioxidant property of RJ makes it a potential scavenger for free radicals. We thought that increasing FRAPS and reduced NO levels in our study might be related to these beneficial effects.

In addition, Ramadan and Al-Ghamdi (24) showed that 29 peptides with antioxidative activity were separated from RJ protein hydrolysate. Albeit, 12 of these antioxidative peptides with 2-4 amino acid residues had the highest free radical scavenging effects. In another study, Silici et al. (33) showed that administration of RJ (100mg/ kg) to cisplatin-treated rats increased antioxidant enzyme activities (SOD), catalase (CAT) and glutathione-peroxidase, while decreasing malondialdehyde levels in their samples. Accordingly, the results of our study showed that RJ administration caused a significant increase in serum FRAP levels in immature female rats. This is in agreement with the findings of previous studies that reported a significant increase in FRAP and antioxidant enzyme activity levels in diabetic male rats treated with RJ (14). Consequently, our data emphasize the antioxidant effects of RJ. We suggest that appropriate administration of RJ may affect female fertility due to its antioxidant and estrogenic effects. Further research is required to evaluate the molecular mechanisms associated with the effects of RJ on female reproductive system, especially prior to using this compound as a treatment for human patients. 

## Conclusion

Administration of RJ to immature female rats promotes follicular growth and development in their ovaries. The mechanism of its action might be through its antioxidant and estrogenic effects on reproductive system to ameliorate the fertility parameters. RJ can potentially be considered as a treatment to promote fertility. 
